# The Multifaceted Roles of Ku70/80

**DOI:** 10.3390/ijms22084134

**Published:** 2021-04-16

**Authors:** Sayma Zahid, Murielle Seif El Dahan, Florence Iehl, Paloma Fernandez-Varela, Marie-Helene Le Du, Virginie Ropars, Jean Baptiste Charbonnier

**Affiliations:** Institute for Integrative Biology of the Cell (I2BC), Université Paris-Saclay, CEA, CNRS, 91198 Gif-sur-Yvette, France; sayma.zahid@i2bc.paris-saclay.fr (S.Z.); murielle.seif-el-dahan@i2bc.paris-saclay.fr (M.S.E.D.); florence.iehl@evotec.com (F.I.); paloma.fernandez-varela@i2bc.paris-saclay.fr (P.F.-V.); marie-helene.ledu@i2bc.paris-saclay.fr (M.-H.L.D.); virginie.ropars@i2bc.paris-saclay.fr (V.R.)

**Keywords:** double-strand break, c-NHEJ, telomeres, protein-DNA interactions, DNA repair machinery

## Abstract

DNA double-strand breaks (DSBs) are accidental lesions generated by various endogenous or exogenous stresses. DSBs are also genetically programmed events during the V(D)J recombination process, meiosis, or other genome rearrangements, and they are intentionally generated to kill cancer during chemo- and radiotherapy. Most DSBs are processed in mammalian cells by the classical nonhomologous end-joining (c-NHEJ) pathway. Understanding the molecular basis of c-NHEJ has major outcomes in several fields, including radiobiology, cancer therapy, immune disease, and genome editing. The heterodimer Ku70/80 (Ku) is a central actor of the c-NHEJ as it rapidly recognizes broken DNA ends in the cell and protects them from nuclease activity. It subsequently recruits many c-NHEJ effectors, including nucleases, polymerases, and the DNA ligase 4 complex. Beyond its DNA repair function, Ku is also involved in several other DNA metabolism processes. Here, we review the structural and functional data on the DNA and RNA recognition properties of Ku implicated in DNA repair and in telomeres maintenance.

## 1. Introduction

Living organisms are constantly exposed to genotoxic stress that can lead to double-strand breaks (DSBs). These DNA lesions can be of endogenous origin, such as cellular respiration and reactive oxygen. They can also be of exogenous origin such as ionizing radiation, pharmaceutical drugs, or chemical exposure [1] DSBs are repaired by two main DNA repair pathways: Homologous recombination (HR) and classical nonhomologous end-joining (c-NHEJ). The c-NHEJ is the prominent DSB repair pathway in multicellular eukaryotes, while HR is predominant in *S. cerevisiae*. The c-NHEJ pathway accounts for most DSB repair outside of the S and G2 phases [2,3].

The Ku70/80 complex was first identified in 1981 by Mimori et al. as a high-molecular-weight nuclear protein and as an antigen recognized by auto-antibodies in sera from patients with polymyositis-scleroderma diseases [4]. It was called Ku from the first two letters of the original patient’s name whose serum was used as a prototype to identify and purify the heterodimer. The heterodimer Ku70/80 (called Ku hereafter) is a central actor of the c-NHEJ pathway. It is considered as one of the first factors to be recruited at DSB sites because of its high affinity for DNA ends (Kd in the nM range) and its high abundance in the nucleus [5]. Ku recognizes a large variety of DNA ends including blunt ends, hairpin DNA, or ends with protruding single-stranded overhangs (discussed in detail later on). Via its *C*-terminal region, Ku interacts with the DNA-dependent protein kinase catalytic subunit (DNA-PKcs) to form the DNA-PK holoenzyme, which controls the interaction between broken DNA ends during c-NHEJ [2,3,4]. Ku has a key role in c-NHEJ beyond the ends recognition step. It directly or indirectly recruits most c-NHEJ effectors, including ends processing by nucleases, kinases, and phosphatases; nucleotide addition by DNA Pol X polymerases; and ligation by the XRCC4/Lig4 complex and the XLF and PAXX scaffolding proteins [5,6]. In this review, we mainly focus on the molecular basis of the interactions of Ku with various DNA ends observed during DSB formation and on its interactions with RNA and DNA substrates at telomeres.

The molecular basis of the protein interactions between Ku and the c-NHEJ factors are reviewed elsewhere [7,8,9]. Briefly, recent structural studies characterized interaction sites of Ku with c-NHEJ proteins. The structure of the DNA-PK, formed by Ku and DNA-PKcs, was determined by cryo-electron microscopy (cryoEM) by several groups (for example, [10,11]). Recently, Chaplin et al. identified particles corresponding to a dimer of DNA-PK holoenzymes that reconstitute a synapse of DNA ends. This dimer is mediated by a domain swap mechanism of the conserved *C*-terminal helix of Ku80. In this dimer, DNA ends present in each DNA-PK run almost parallel [11]. Ku can also recruit, at DSBs, several c-NHEJ factors containing motifs called Ku-binding motifs (KBMs) [12,13]. These KBMs were identified in the nuclease APLF, the ligation factors XLF (XRCC4-like factor) and PAXX (PAralog of XRCC4 and XLF), the regulation factor MRI/CYREN (MRI for Modulator of Retrovirus Infection, CYREN for Cell Cycle Regulator Of Nonhomologous End Joining), and the helicase/nuclease WRN protein [12,13]. Additional direct protein interactions were also reported with the Pol X polymerases, pol mu and TdT, and with the LIG4/XRCC4 complex [12]. Ku is thus a major hub of the c-NHEJ with key functions in DSB recognition and in coordination of the c-NHEJ enzymatic activities.

## 2. Ku70/80, an Abundant Nucleus Factor with High Affinity for DNA Ends

In human cells, Ku70 (69.9 kDa, 609 aa) and Ku80 (82.7 kDa, 732 aa) assemble to form a heterodimer The role of Ku in DNA repair was first established experimentally when it was discovered that the Ku80 subunit was defective in X-ray-sensitive rodent cell mutants in the XRCC5 group (X-ray repair cross-complementing group 5) [7,8,9]. The same observation was made in embryonic stem cells XRCC6 defective for Ku70 [14]. The Ku70/80 heterodimer is one of the most abundant nuclear factors (about 500,000 copies by cell), and this corresponds to a cellular concentration of about 1.5 µM if we consider a nucleus with a radius of 5 µm. The Ku70 and Ku80 subunits are mainly addressed to the nucleus through the presence of a nuclear localization signal (NLS) present in the *C*-terminal region of each subunit (NLS in position (539–556) in Ku70 and in position (561–569) in Ku80) [15,16]. The crystal structures of the Importin α in a complex with the NLS of Ku70 or Ku80 were reported [17]. Both NLSs function as monopartite NLSs with only one basic cluster in the interaction with the importin. Mutagenesis of the NLS showed that each subunit can be imported via its own NLS or the NLS in the other subunit [18]. Ku is also present in the cytoplasm and at the membrane where it exerts several functions, including an anti-apoptotic activity by sequestering Bax (see [19] for a review).

In 1986, Mimori et al. unveiled in a seminal study the basic characteristics of the Ku protein regarding its DNA binding properties [20]. After the purification of Ku from HeLa extracts by immunoabsorbent column chromatography, they showed that Ku binds double-strand DNA (dsDNA) more efficiently than single-strand DNA (ssDNA), and they identified an interaction with dsDNA that is salt-dependent. They reported that Ku efficiently binds a linearized plasmid but not the circular form. Using restriction enzymes, they observed that the interaction is proportional to the number of cuts and is independent of the sequences at the cleavage sites. From all these data, they proposed that Ku interacts tightly with DNA ends in a sequence-independent manner [20]. The authors made the hypothesis that Ku may be “associated with DNA break points that might occur through the action of such agents as UV light or certain drugs” and thus have a role in DNA damage repair.

## 3. Affinities of Ku for DNA Measured by Biochemical and Biophysical Approaches

Several additional studies further documented the DNA binding properties of Ku. The interaction of Ku with short dsDNA (in the 20–200 bp range) was measured by different approaches. Initially, most studies used EMSA (electrophoretic mobility shift assay), where the interaction between Ku and DNA results in characteristic “ladder” profiles. Different species with reduced mobilities form a ladder, with each band corresponding to a different number of Ku molecules bound on the DNA used. The EMSA assays indicate that one Ku molecule is loaded for every 30 bp of a DNA duplex and that Ku can translocate internally, leaving a place for new Ku molecules at the ends [21]. The first electron microscopy analyses of Ku on DNA confirmed the ability of Ku to thread on DNA and fully cover a DNA duplex [22].

The apparent Kd measured by EMSA with purified Ku and short dsDNA fragments (16–24 bp) ranged from 30 pM to 2 nM according to the experimental conditions [21,23]. The dissociation constants were further characterized using double-filter determination and anisotropy fluorescence [24]. Similar Kd values were measured in the 4–7nM range. Recently, we used calorimetry to measure the interaction between Ku and different DNA substrates [25,26,27]. We obtained dissociation constants of 3.5 and 2.4 nM between Ku and two DNA duplexes of 18 and 42 bp, respectively, in good agreement with the previous values reported. The calorimetry gives access to the stoichiometry of the interaction. By titrating Ku with the 18 and 42 bp, we measured DNA:Ku stoichiometries of 0.97 and 0.47, respectively, which is in good agreement with the one and two Ku molecules bound on these substrates, respectively. Ku presents a slighter preference for DNA ends that are A/T-rich compared to G/C-rich, suggesting that some unwinding of the DNA ends may facilitate the interaction [23].

A helicase activity was initially proposed for Ku [28] with heterodimers purified from HeLa cells or *E. coli*. The absence of the motor domain and ATPase site in Ku suggests that it could correspond to a contaminant helicase co-purified with Ku. Indeed, Ku exhibits some affinity for ss oligos but in a much lower affinity range than for dsDNA [23,29,30]. Nevertheless, high concentrations of ssDNA can divert Ku from DSBs and favor Ku-independent end-joining at the expense of c-NHEJ [30]. Ku binding to single-stranded DNA (ssDNA) requires a free DNA end, a minimal length of 10 nucleotides with an optimal length of 25 nucleotides, and a preference for ssDNA with a free 5′-end [30]. Ku can also bind ssDNA once, forming a hairpin structure [31].

Ku70 and Ku80 are predominantly observed as a heterodimer when expressed in *E. coli* or in insect cells. Wang et al. showed that purified Ku70/80 and Ku70 expressed in rabbit kidney cells (RK13) bind dsDNA with a similar affinity and showed a similar sensitivity to high-ionic-strength buffers [32]. More recently, homodimers of Ku70 were isolated from the expression in *E. coli* and as a byproduct of the purification of Ku from insect cells [33]. The Ku70 homodimer stably binds 50 bp dsDNA substrate-forming complexes observed as two bands with an apparent lower affinity than the Ku heterodimer [33]. Ku70 and Ku80 subunits were separately expressed in vitro using rabbit reticulocytes lysate, and no DNA binding was observed with these isolated subunits [34].

## 4. Kinetic Analyses of Ku Binding on DNA

Kinetic analyses with DNA of increasing length (18 to 90 bp) were reported by Ma et al. using SPR (surface plasmon resonance) [35]. The authors showed that Ku binds a short DNA (18 bp) with a fast association rate (kon) of 7.2 × 10^6^ M^−1^s^−1^ and a dissociation (koff) rate of 1.0 × 10^−2^ s^−1^, corresponding to a Kd of 1.4 nM. Similar kinetic constants were reported with Ku from *S. cerevisae* using SPR [36]. A cooperative binding was observed for two sites (45 bp), suggesting that the first Ku molecule positioned at the DNA end favors the recruitment of the second Ku molecule. The use of DNA with 3 and 4 sites (68 and 90 bp) does not show additional cooperativity. Recently, some kinetic data were reported using SPR and the differences in Förster resonance energy transfer (FRET) with Ku70 and Ku80, respectively, tagged with an ECFP or EYFP protein in their *N*-terminus [37]. The SPR results were in the same range than previous measurements. Using FRET, a longer time release was observed with a k_off_ of 1.4 × 10^−4^ s^−1^, and the authors observed that a deletion mutant of the SAP domain (SAF-A/B, Acinus, and PIAS) of the Ku70 Cter region rapidly dissociates, resulting in a 20-fold reduced affinity for DNA [37].

## 5. Ku Recognizes a Large Variety of DNA Ends

**Figure 1:**
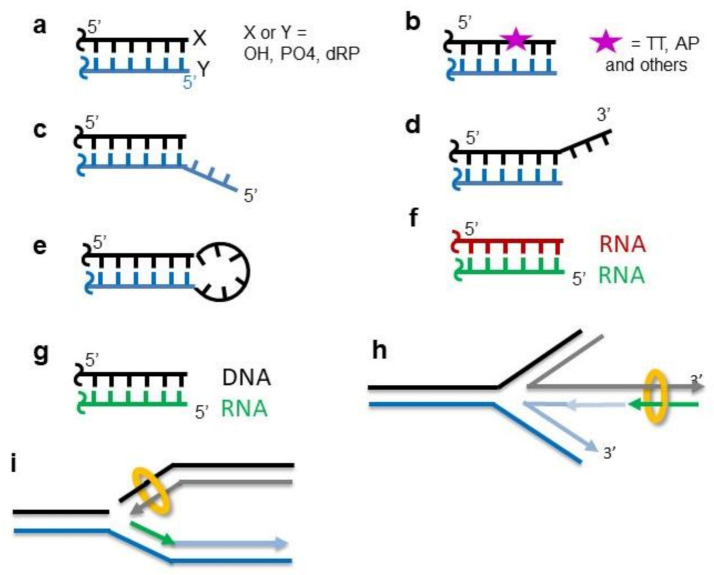
Diversity of DNA ends that are recognized by Ku70/80. (**a**) Ku binds DNA ends efficiently with strands containing either hydroxyl or phosphate functions, as well as more heterogeneous ends with dRP or other chemical adducts. (**b**) Ku recognizes DSBs with a lesion present in the internal position close to the DNA ends. (**c**,**d**) Ku interacts efficiently with DNA having single-strand overhangs in 3′ and/or in 5′ up to 20 nt. (**e**) Ku can accommodate the presence of loops up to at least 8 nt at the ends. (**f**) Ku binds RNA hairpins at telomeres. (**g**,**h**) Ku interacts with the DNA–RNA duplex during replication fork restart (nascent RNA (green arrow)). (**i**) Ku binds single-ended DSB.

An important issue regarding the DNA ends recognition by Ku is the capacity of the heterodimer to recognize a wide spectrum of DSBs with different DNA ends structures. DSBs are generated by various endogenous and exogenous agents that result in chemically heterogeneous structures and chemical groups at DNA ends (reviewed in [38]) (Figure 1a). Using restriction enzymes generating blunt or 5′-or 3′-overhang DNA ends, Mimori et al. showed that the affinity of Ku is independent of the presence of 3–4 nucleotides overhangs [20]. This was confirmed by Falzon et al., who observed a tight interaction with a DNA containing an overhang of 21 nt on the four ends of a dsDNA [23]. Tadi et al. observed that Ku can accommodate DNA large single-strand overhangs in 3′ or 5′ [33] (Figure 1c,d). Ku can also accommodate hairpin structures (Figure 1e). Affinities similar to blunt DNA were measured with DNA containing hairpins of 4 to 20 nucleotides at both ends [23,24]. It is still unclear how the Ku ring structure can adapt to this variety of DNA ends.

Ku also interacts with DNA ends, presenting DNA damage at proximity. Turchi et al. showed that Ku can bind a 75 mer DNA treated with Cisplatin (CisPt) (Figure 1b), although under this condition, it does not activate DNA-PKcs [39], contrary to what is observed in the absence of DNA damage close to the DSB ends [40]. Ku interacts with a DNA duplex containing an abasic site close to the DNA ends, and it was identified as the main protein trapped with the crosslinking reagent and DNA containing AP sites in the cellular extract [41]. In agreement with this observation, 5′dRP/AP lyase activity was reported for Ku. This activity is proposed to “clean” AP sites located close to DSBs and, thus, contribute to the DSB processing [42]. Ku lyase activity has a preference for the AP site located on a 5′ overhang. Mutations of three Ku70 lysines (K31, K160, and K164) located on the inner part of the ring lead to a major reduction of this activity [43]. The AP lyase activity was also characterized in two bacterial Ku proteins [44].

## 6. A Unique Pre-Formed Ring Structure among DNA Binding Proteins

**Figure 2:**
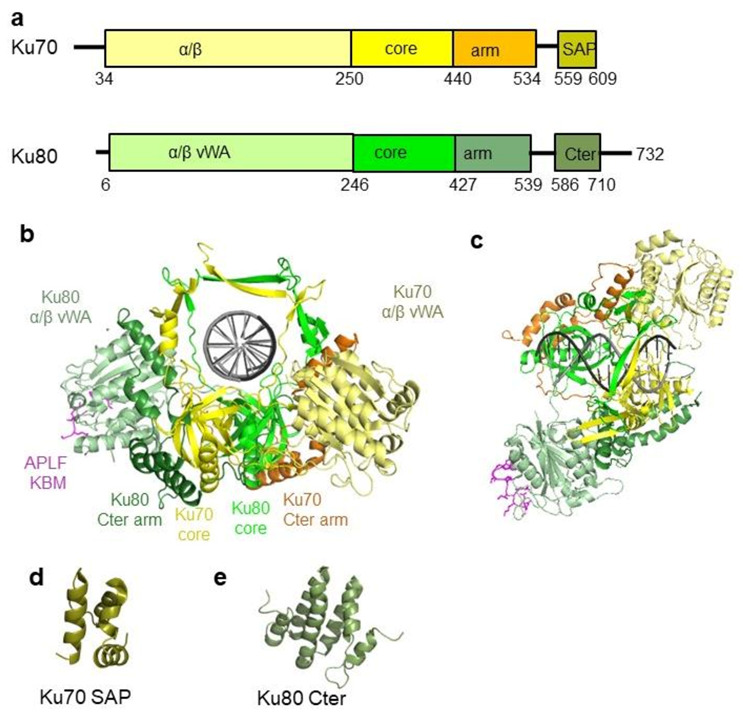
Structure of the human Ku70/80 heterodimer. (**a**) Human Ku70 and Ku80 have similar organizations. They present some sequence variabilities in their *C*-terminal region. (**b**,**c**) Crystal structure of Ku70/80 complexed with a hairpin DNA and the Ku binding motif of APLF (magenta) [26]. Ku is colored in the same way as that in (**a**). The right view shows the top view of the complex. The DNA is a hairpin DNA used to limit Ku movement on the DNA for crystallization. (**d**) Structure of the SAP domain of Ku70 solved by NMR [48]. (**e**) *C*-terminal domain of Ku80 solved by NMR [49,50].

A major advance in the understanding of the Ku mechanism was the crystal structure of the heterodimer in the complex with hairpin DNA in 2001 [45]. A key observation was the ring-shaped structure of the heterodimer surrounding the DNA (Figure 2). The crystal contained a DNA with a 14 bp duplex region followed by a 13 nt hairpin to limit Ku translocation during complex formation and crystallization. The protein was formed by three domains: An *N*-terminal α/β domain, a central-barrel domain, and a helical *C*-terminal arm (Figure 2). Ku70 and Ku80 have a C-terminal region (SAP domain (7 kDa) in Ku70 and a Cter of 17 kDa in Ku80). The Ku70 SAP domain is present in both crystals of Ku free (apo-Ku) and Ku complex, but it is only visible in the crystal structure of apo-Ku where it is in contact with the Ku80 α/β domain. Using electron microscopy, Rivera-Calzada mapped these *C*-terminal regions and their conformational changes after DNA and DNA-PKcs binding on maps at low resolution [46]. Recently, origami DNA were used to visualize, by EM, one or two Ku molecules bound at DNA ends specifically positioned on the origami [47].

The Ku ring is formed by a large base that cradles DNA and a narrow bridge formed by β-strands from each subunit (Figure 2b,c). The *N*-terminal α/β domains are positioned at a remote distance from the ring structure. The central rounded β-barrel domains of Ku70 and Ku80 contribute to the dimer interface, and each contain a ~70 residues insertion that forms the two narrow bridges and the ring-shaped structure of Ku. The extremities of these insertions are buried in the Ku70 and Ku80 subunits so that their opening is highly unlikely and would need a large unfolding of the Ku subunits. Ku has an asymmetric orientation on DNA ends. Yoo et al. used cross-linking experiments and characterized a preferential orientation of the heterodimer. The Ku70 subunit is located proximal to the DNA end, and Ku80 is located toward internal DNA nucleotides [51]. The crystal structure thus reveals a preformed ring structure that fits well with the specific DNA ends recognition properties of Ku. Ku covers the 14 bp duplex in the crystal structure, but molecular modeling from this structure suggests that about 25 bp could bind to the large Ku cradle. This ring structure with two interdigitated insertions raised the question of the unloading of Ku after DNA ligation and was recently addressed (see below).

## 7. The Ku70/80 Has an Apparently Rigid Inner Face

**Figure 3:**
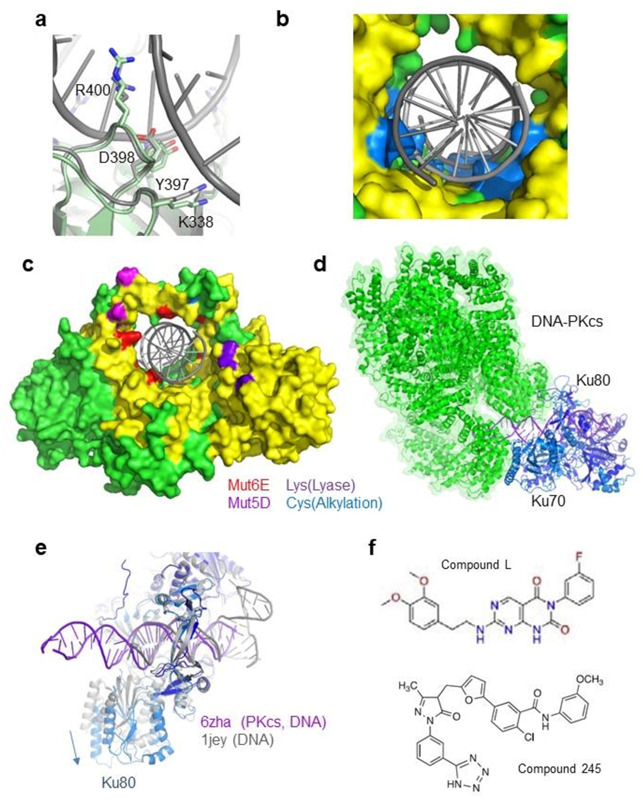
Complementarity between Ku inner ring and the DNA (**a**) Structure of an inner ring loop of Ku (Ku80 Tyr397-Arg400) that interacts with the minor groove of the DNA. The figure shows a superimposition of this loop in the Ku/DNA complex and Ku free structures. (**b**) Surface representation of the Ku inner ring. Ku residues in contact with the DNA grooves are colored in blue. Due to these contacts, the radius of the inner ring of Ku is smaller than the DNA radius. (**c**) Surface representation of Ku. Residues in red are the six Ku70 positions mutated in Glu to disrupt the Ku-DNA interaction [52] (positions Lys282, Lys287, Thr300, Lys331, Lys338, and Arg403). Residues in magenta are the five positions mutated in Asp in [53] to mimic potential phosphorylation sites on the ring (Thr305, Ser306, Thr307, Ser314, and Thr316). Residues in purple are positions of Ku70 lysines (Lys31, Lys160, and Lys164) proposed to be involved in the Ku dRP/Lyase activity [43] (Lys31 is not observe, the first amino acid of Ku70 visible in the crystal is Arg35). Residues in light blue are a Cys of Ku80 that is a potential site of alkylation [54]. (**d**) CryoEM structure of (Ku70/80)/DNA-PKcs/DNA complex (PDB 6zha) [11]. DNA-PKcs is represented in the green cartoon with a semi-transparent surface. Ku70 and Ku80 are, respectively, shown in light and dark blue, while DNA is shown in purple. (**e**) Superimposition of Ku from the Ku/DNA-PKcs/DNA complex and Ku from the Ku/DNA complex (PDB 1jey) [45]. The loops of Ku in contact with DNA superimpose well, indicating that Ku has the same inner ring conformation with or without DNA-PKcs. (**f**) Inhibitors of Ku-DNA interactions reported by [55] (compound L) and by [56] (compound 245).

The crystal structure of the Ku/DNA complex shows that several loops of Ku70 and Ku80 are in contact with the minor and major grooves of the DNA. The inner dimension of the Ku ring is thus smaller than the external radius of the DNA helix, similar to a screw fixed in a nut (Figure 3a,b). These loops superimpose well in the apo structure of Ku or in a complex with the DNA. This suggests a limited dynamic of these loops that are not engaged in crystal contacts (Figure 3a). In recent crystal and cryoEM structures of the Ku-DNA complex bound with DNA-PKcs or peptides [10,11,26], these loops also adopt the same conformation, suggesting a stable conformation of these loops with or without DNA. It remains to be documented if some rearrangements can happen during Ku translocation or if Ku threads in a helical manner like a screw on a nut.

Mutations of the Ku ring have been characterized with the aim to disrupt the interaction between Ku and DNA. Britton et al. thus designed a mutant called Mut6E with six residues of the Ku70 ring mutated in Glu to induce a repulsive charge (K282E K287E T300E K331E K338E R403E) [52] (Figure 3c). The Mut6E mutant does not bind DNA anymore and was used as a control in a super-resolution analysis of Ku localization.

## 8. Insights from Recent 3D Structures of Ku-DNA Complexes Bound with C-NHEJ Factors

Recently, crystal structures of the Ku70/80/DNA complex were reported bound with peptides derived from Ku-binding motifs (KBM) of the nuclease APLF and of the ligation factor XLF [12,26]. The binding site of the KBM of APLF (A-KBM) is located at the periphery of the vWA (von Willebrand factor type A) domain of Ku80 in a position remote from the DNA binding site (Figure 2b). The amino acids involved in the interaction with DNA superimpose well in the Ku-DNA-(A-KBM) and in the Ku-DNA structures [26]. In agreement with this observation, the affinity of Ku for dsDNA is the same with or without an A-KBM peptide. The KBM of XLF (X-KBM) is positioned in a remote site that is created after a large opening of the vWA domain that creates a binding pocket for this motif. The X-KBM is located closer to the DNA than the A-KBM is, but its binding has no impact on the affinity of Ku for a dsDNA. It is unknown if the other regions of the XLF and APLF can bind on Ku or DNA and if they have an impact on the Ku affinity for DNA or on its ability to translocate on DNA.

Several crystals and cryoEM structures of DNA-PKcs free and in a complex with Ku were reported [10,11,57,58,59,60]. Two recent cryoEM structures reported at a higher resolution used Y-shaped DNA with a 35 bp duplex region [10,11] (Figure 3d). In these structures, Ku interacts with DNA in a similar manner than in the crystal structures. The authors observed that the Ku70 and Ku80 subunits superimpose well with their counterparts in the absence of DNA-PKcs, except a slight movement of the α/β domain of Ku70 (Figure 3d,e). What is noteworthy is that the interactions of Ku with DNA-PKcs and the KBM peptides do not impact the position of the Ku loops that define the inner ring of the heterodimer. This observation favors the hypothesis of a conserved preformed state of the inner ring structure of Ku in different contexts.

## 9. Ku Can Recognize RNA Hairpins and RNA-DNA Hybrids

Ku was shown to bind about 200–500-fold less efficiently DNA–RNA hybrids such as poly(A)-poly(dT) or yeast tRNA, suggesting a marked specificity for dsDNA [20]. Another study by Kaczmarski et al. showed that Ku binds HIV-1 TAR RNA that forms a sTable 50 nt hairpin structure with a 10-fold weaker affinity than dsDNA [61]. Using the SELEX approach (Systematic Evolution of Ligands by Exponential Enrichment), a series of RNA aptamers was characterized by Yoo et al. with tight affinities (Kd ≤ 2 nM), similar to the Kd observed with dsDNA [62]. Another study by Anisenko et al. suggests that Ku70 without Ku80 can bind dsDNA and hairpin RNA with good affinities (Kd of 60 nM for dsDNA and 40 nM for hairpin RNA) [63]. A crystal structure of a complex, the Integrator complex with subunits 13 and 14 (INTS13/INTS14), with a structure reminiscent of the Ku heterodimer, was recently reported [64]. This complex processes 3′-ends of the spliceosome small nuclear RNAs (snRNAs). It presents structural similarities with Ku70/80, despite its low sequence similarities (identity 7–10%, similarity 17–19%). Ku70/80 and INTS13/INTS14 share highly similar domain architectures with *N*-terminal vWA domains, central β-barrels, and *C*-terminal α-helical domains. The INTS complex can bind single- and double-stranded DNA and RNA.

## 10. Ku Contributes to Synapse DNA Ends in a Complex with Other C-NHEJ Factors

An important issue in the c-NHEJ pathway is to maintain the DNA ends in close proximity and form a synapse between them. This is central to limit the ligation of one DNA extremity with the DNA end of another DSB and, thus, prevent chromosome translocation. Ku has a central role in the synapse formation all along the c-NHEJ pathway. It coordinates the alternative recruitment of the different c-NHEJ enzymatic activities on the DSB ends and passes on the baton from one to another without leaving DNA damage. This function is reminiscent of the role of factors of other DNA repair pathways like XRCC1 in BER (base excision repair) or MutS in MMR (mismatch repair) that stay at the site of damage, recruit downstream factors, and limit the release of DNA until the repair is complete [65]. In the first steps of c-NHEJ, the main synaptic activity is attributed to DNA-PKcs in a complex with Ku [66]. However, Zhao et al. showed with single-molecule FRET (smFRET) that both Ku and XRCC4:DNA ligase IV are necessary and sufficient to achieve a flexible synapsis of blunt DNA ends, whereas either alone is not [67]. Indeed, by itself, Ku has a weak synaptic activity between two DNA ends. Ramsden et al. also observed with an elegant assay using a biotin-DNA and a ^32^P-labelled-DNA that in the presence of Ku, a fraction (8%) of labelled-DNA is captured on streptavidin beads [68]. Similarly, Wang et al. showed with magnetic tweezers experiments that Ku by itself cannot form a synapse between two DNA ends [69]. They observed a transient synapse in the presence of Ku and DNA-PKcs with a half-life of 0.1 s. The addition of PAXX and Lig4/XRCC4/XLF reinforces the stability of the synapse up to 66 s in this system. In *S. cerevisiae*, no DNA-PKcs has been identified so far [70]. The MRX complex (Mre11/Rad50/Xrs2) has an important role in c-NHEJ and it is proposed to contribute to synapse formation as observed by AFM (atomic force microscopy) [71,72].

## 11. Number of Ku Molecules at the Ends: In Vitro versus Cellular Analyses

In vitro studies showed that several Ku molecules can thread on dsDNA with up to one Ku loaded every 25–30 bp. The first Ku molecules bind close to DNA ends and then translocate inward so that other Ku molecules can have access to the ends. An EMSA performed with Ku thus presented regular ladder profiles where the different bands correspond to an increasing number of Ku molecules on DNA. Biophysical measurements with calorimetry [26], anisotropy fluorescence [73] and SEC-MALS (size-exclusion chromatography with multiangle light scattering), SPR, or BLI (bio-layer interferometry) (unpublished data) are in agreement with this mechanism. The molecular mechanism of the translocation of Ku is not coupled to ATP hydrolysis as for translocases and helicases. From the structural studies, we can suggest two hypotheses. As described above, the inner part of the Ku ring makes tight contacts with the minor and major grooves of DNA. One possibility is that the ring presents some conformational changes on the loops in contact with the DNA and somehow enlarge to allow a passive one-dimensional diffusion of Ku on the DNA. A second hypothesis is that the inner ring has a rigid and stable conformation so that Ku translocate with a helical translation (Figure 3b).

The translocation of Ku on DNA has been proposed to be more limited in a cellular context by using high-resolution microscopy. A seminal study used ribonuclease- and detergent-based pre-extraction treatment to improve the visualization of Ku at DSB ends [52]. Ribonuclease treatment favors the release of a large proportion of Ku molecules bound to nuclear structures through interactions with RNA. This method combined with STED (stimulated-emission-depletion) microscopy allowed the visualization of Ku foci of small spherical shape (size of about 80 nm), corresponding to a maximal number of three to four molecules at each DNA end. Analyses by bleaching steps of fluorescent Ku molecules suggest that, predominantly, only one Ku molecule may be present on each side of the DSB [52]. These results indicate that despite a high concentration of Ku in the cell, the threading of Ku is reduced in the cell environment by some factors or processes.

## 12. Factors That May Limit Ku Threading in Cell

**Figure 4:**
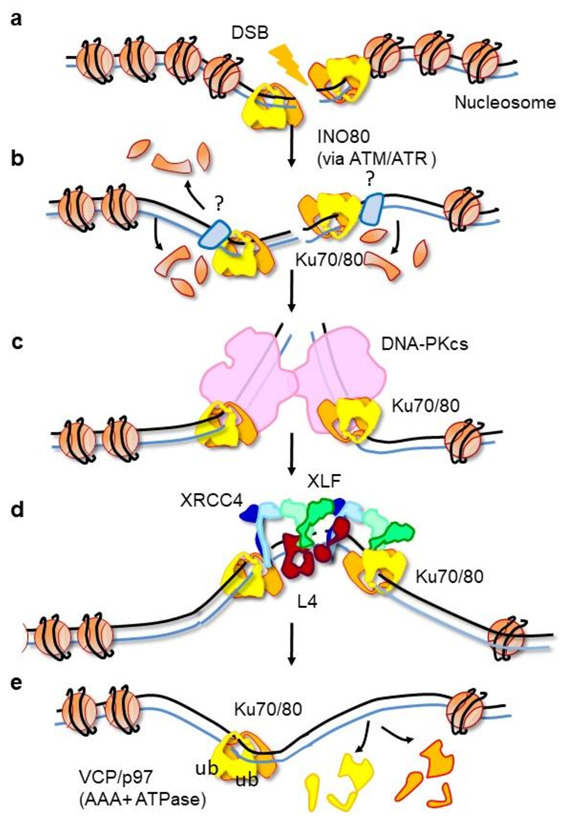
Association and removal of Ku in a chromatin context. (**a**) Upon DSB formation, Ku recognizes DNA ends. Super-resolution microscopy studies suggest that only one Ku may bind at the DSB and that no threading inward is observed with the loading of additional Ku molecules [52]. (**b**) Upon DSB formation, some nucleosomes are disassembled by the nucleosome remodeler INO80 [75]. Some factors not yet identified likely limit the Ku threading inward (light blue protein). c-NHEJ factors like XLF, APLF, or PAXX may be involved in this role. (**c**) Ku recruits DNA-PKcs, and the complex forms a synapse between the two DNA ends in a conformation that may correspond to the dimer observed by Chaplin et al. [11]. (**d**) Ku can recruit alternatively nucleases, polymerases, and the ligation complex at the DSB ends (here, the Ligase 4 is represented in a complex with an XLF-XRCC4 filament [104,105]. (**e**) Once the ligation is completed, Ku is trapped on the DNA. The AAA+ ATPase VCP/p97 will remove in place the trapped Ku molecules [101].

One hypothesis regarding the limited threading of Ku in the cell is the presence of nucleosomes at DSB ends that may block the Ku diffusion. In vitro studies with EMSA showed that Ku efficiently binds DNA ends close to the nucleosome, though with reduced apparent affinity (apparent Kd of 0.3 and 6 nM on free dsDNA and DNA ends, respectively, close to the nucleosome) [74]. In this study, the authors showed that Ku is able to displace the protein linker histone H1 from DNA, but it does not displace the nucleosome. They proposed that Ku “peels” some 50 bp DNA from the nucleosome [31,74]. The dynamic of nucleosomes following the formation of a DSB was analyzed using the I-PpoI homing endonuclease and the ChIP (chromatin immunoprecipitation) analysis of histone H3 around the I-PpoI break sites [75]. The authors observed a decrease in H3 over 1000 bp, corresponding to about eight nucleosomes (146 bp of DNA are wrapped around an octamer of histone H2A, H2B, H3, and H4). The disassembly is promoted in an ATM- and ATR-dependent manner by the nucleosome remodeler INO80 [75] (Figure 4). However, the extent of this nucleosome clearing would allow more Ku sliding than observed in the cells [52]. A second hypothesis is that other DNA binding proteins may limit Ku diffusion inward through interactions with a proximal Ku molecule and adjacent DNA. c-NHEJ factors interacting with Ku with DNA binding properties may play such a role. These include DNA-PKcs, XLF, APLF, WRN, Lig4/X4, PAXX, and likely MRN (Mre11/Rad50/Nbs1) [12,13,76,77,78].

## 13. The Race for the DSB Ends

The two main DSB repair pathways are homologous recombination (HR) and nonhomologous end-joining (NHEJ). HR requires a homologous template to guide the repair of the broken DNA and, therefore, functions in S and G2 phases due to the presence of a sister chromatid, whereas c-NHEJ is active during all cell cycles out of mitosis and mediates the direct re-ligation of the broken DNA. The complexity of the broken ends can determine the choice of the repair pathway [5]. Simple DSB ends are mainly repaired by c-NHEJ, and complex DSBs often require end resections. c-NHEJ requires limited sequence homologies (0–4 bp) between the overhangs of the DNA ends, while HR requires extensive end resection with a typically long homology tract (more than 100 bp). Several factors influence the choice of the pathway including the cell cycle stage, the DNA end resection, and specific post-translation modifications. At least four sensors detect DSB: Ku, PARP, MRN, and RPA after DSB processing. Using a pulsed near-infrared (NIR) laser, Mari et al. showed that Ku starts localizing at DSB within a few seconds and reaches a maximum after 3 min, and that 20% remains after 2 h [79]. Another laser-induced study showed that not only Ku but also MRE11 and PARP-1 are recruited immediately and independently to damage sites [80]. These factors are still detectable on DSB for about 2 h for PARP1, 4–6 h for Ku, and more than 8 h for MRE11. A meta-analysis of recruitment data of over about 80 DSB-related proteins was reported [81] (http://www.dna-repair.live/cumulus/, accessed on 17 March 2021). From this study, c-NHEJ factors are among the first to arrive at the DSB sites followed by the slower HR machinery. The median time for access on the DSB is about 10 s for Ku. It is among the fastest with c-NHEJ factors like Artemis, XLF, and PNKP (polynucleotide kinase 3′-phosphatase). PARP-1 (Poly (ADP-ribose) polymerase-1) belongs to the rare factors that are reported to be faster (about 3 s). This is in agreement with previous studies that report a fast access of PARP-1 at DSB [82]. In yeast, different studies showed that Ku and MRX/MRN are among the first proteins recruited to DSB [72,83,84,85].

Besides the competition to be first place, sensors can be actively removed. In budding yeast, meiotic DSB processing gave some clues to the removal of Ku by the combined activity of the Mre11-Rad50-Nbs1 complex (MRN) and CtIP (Ctp1/Sae2) required to initiate DSB resection [86,87]. It was shown that Sae2 (CtIP in humans) activates Mre11 endonuclease activity at a distance from the Ku-DNA ends and that this nick serves as at entry point for exonucleases [86]. This mechanism was confirmed by biochemical and single-molecules analyses [88,89,90], showing that Ku can be removed by the activity of these combined nucleases. It is well established that Ku prevents the exonucleolytic attack of free DNA ends [84,91,92,93]. In cells, Ku is also a major inhibitor of an alternative end-joining pathway involving an MRN-dependent ends resection step [94].

## 14. Ku Molecules Trapped after Ligation Are Actively Removed

The Ku ring has no clasp, and it is unlikely to open to dissociate. The crystal structure shows a large intertwining of both subunits (Figure 2b). An opening would require an unwinding of the *C*-terminal arms of Ku70 and Ku80 (constituting 95 and 113 amino acids, respectively). The ring structure of Ku is different from other ring shape factors with DNA binding properties like the PCNA trimer (proliferating cell nuclear antigen). The PCNA is a trimeric protein involved in the processivity of the replicative DNA polymerase. It can assemble and disassemble on dsDNA independently of the presence of DNA ends with the help of the replication factor C [95]. Paillard et al. showed in vitro that Ku is stable when trapped after ligation on mini-circles DNA. It remains bound in the presence of an increasing amount of NaCl from 0.1 to 2 M under conditions where it dissociates from linear DNA [31].

Different studies recently reported that Ku trapped after ligation would be removed after ubiquitination. Postow et al. showed that Ku80 is degraded in response to DSB in a ubiquitin-dependent manner, but independent of the proteasome [96]. The extraction of Ku from repaired DSB can take place after ubiquitination mediated by three different E3 ligases (SCFFbxl12, RNF8, or RNF138) [96,97,98,99,100]. After ubiquitination, Ku is removed by an AAA+ ATPase called Valosin-containing protein VCP/p97 with its co-factors Udf1-Npl4 [101]. This hexameric AAA+-type ATPase targets ubiquitinated proteins with the help of ubiquitin adaptor proteins. These ATPase use the energy of ATP hydrolysis to segregate their substrate proteins away from their cellular partners or structures (Figure 4e). In U20S cells, Ku80 foci formed after ionizing radiation are largely resolved within 60 min, whereas in the presence of p97 inhibitor, the Ku80 foci persist later [101]. Recently, Ku70 was shown to be a direct target for the deubiquitinase USP14, leading to a reduced recruitment to DSB sites [102]. The conjugation of the ubiquitin-like protein NEDD8 to target proteins at breaks sites has been shown to be required for Ku ubiquitination and Ku and associated repair factors released from these sites following repair [103].

## 15. Ku at Single-Ended DSB and at Stalled Replication Fork

A frequent source of DSB in cells comes from the encounter of the replisome with a discontinuity in the DNA template that generates single-ended double-strand breaks (seDSB) [106] (Figure 1i). With the lack of a second DNA end, the damage cannot be repaired by c-NHEJ and is, therefore, processed by HR in the S/G2 phase of the cell cycle. Ku can bind at seDSB and, thus, needs to be removed to allow resection so that HR can proceed. MRN and EXO1 carry out an incision and excision upstream of the seDSB and remove Ku at the seDSB generated by CPT during replication [107,108]. Balestrini et al. performed a genetic screen in yeast to isolate yKU70 alleles that phenocopy the Ku deficiency with respect to single-ended DSB, but remain proficient for c-NHEJ [109]. They isolated six single mutants that present a reduced affinity for DNA ends and an enhanced capacity to translocate on DNA, as observed by AFM. MRN-Ctp1 also promotes Ku removal after an initial resection in the context of terminally arrested forks. Recent studies in fission yeast show that the presence of Ku at single DNA ends regulates the extension of resection, the level of recruitment of RPA and Rad51, and the efficiency of the fork-restart process (Figure 1h) [110].

## 16. Ku at Telomeres in Yeast

**Figure 5:**
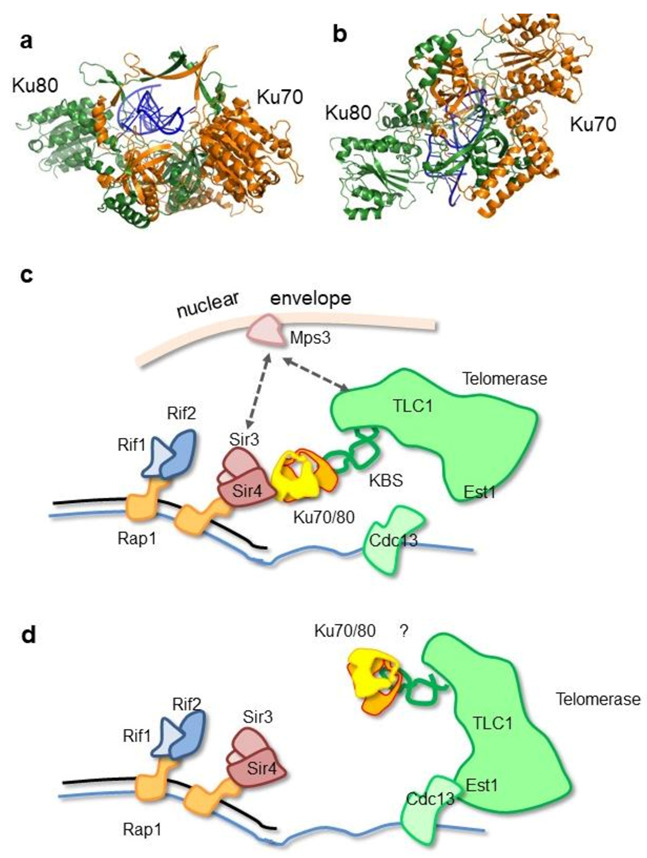
Ku contributes to the recruitment of the telomerase. (**a**,**b**) Crystal structure of *S. cerevisae* Ku70/80 with the Ku binding site (KBS) of the TLC1 RNA of the yeast telomerase [122]. The KBS hairpin RNA is positioned in the ring channel of Ku in agreement with the competition observed between RNA and dsDNA binding. (**c**) Ku is proposed to be recruited at telomeres through its interaction between the Ku80 α/β domain and Sir4 KBM. Ku will contribute to the telomerase recruitment through its interaction with the KBS of TLC1. The interactions of Sir3 and TLC1 with the Mps3 factor at the nuclear envelope is indicated. (**d**) In a second step, the telomerase would interact with Cdc13 to adopt its active state conformation. It is not known whether Ku keeps interacting with TLC1 at this stage (indicated by a “?”).

Telomeres have evolved to protect chromosome termini against degradation, checkpoint activation, and illicit repair [111]. In addition to telomere proteins, Ku70/80 is recruited constitutively at telomeres in budding yeast *S. cerevisiae* [112,113]. Ku directly binds the telomere dsDNA end [114] and protects telomeres against 5′ resection [115,116,117,118]. To prevent Ku-dependent telomere fusions, the Ku function in NHEJ is blocked at telomeres by telomere factors (namely Rap1, Rif2, and Sir4) (Marcand et al., 2008, Lescasse et al., 2013, Roisné-Hamelin et al., 2021). Ku also participates in telomerase recruitment through its interaction with the TLC1 RNA subunit of telomerase and the telomere factor Sir4 [112,113,119,120,121]. The TLC1 RNA region that interacts with Ku is called the KBS for the Ku binding site. The TLC1-KBS specifically encompasses nucleotides 288 to 312 and forms a high-affinity interaction with Ku70/80 (Kd = 75 nM). The crystal structure of TLC1-KBS in the complex with full-length Ku70/80 was recently reported [122]. It reveals that TLC1 adopts a bulged stem-loop structure, and a bent conformation between the P1 and P2 helices of TLC1-KBS upon interaction with Ku [122] (Figure 5).

A direct interaction between the silencing factor Sir4 *N*-terminal KBM motif (100-115) and Ku80 recruits the Ku-telomerase complex to telomeres (Bertuch and Lundblad 2003 MCB) (Chen et al., 2018). Sir4 KBM forms a short alpha-helix that binds a hydrophobic groove inside the Ku80 vWA domain [122] The authors proposed a model where, in the first step, Ku would interact with Sir4 at the telomeres and favor the telomerase recruitment in an inactive manner throughout the cell cycle. In the second step, the telomerase would interact with the ssDNA-bound cdc13 protein, in an active manner, a form competent for telomere elongation [122]. Thus, TLC1 and DNA binding to Ku is mutually exclusive.

As Sir4 is a core component of yeast heterochromatin (Faure et al. 2019 GBE), the KU-Sir4 interaction also favors heterochromatin interactions at telomeres (Ribes-Zamora et al. 2007 NSMB). This pathway is proposed to require a direct loading of KU to the chromosome DNA end (Lopez et al., 2011). Finally, Ku anchors telomeres at the nuclear periphery though Sir4-dependent and -independent pathways [119,120,123,124,125].

## 17. Ku at Telomeres in Mammalian Cells

The organization and functions associated with mammalian telomeres are different from budding yeast (no telomeric silencing or nuclear anchoring), but the Ku heterodimer is also present at the mammalian telomeres [126]. In human cells, Ku is essential to telomere stability and, therefore, cell viability [127]. It has been observed that Ku localizes to telomeres through its high affinity for the TRF1 shelterin protein (Kd = 0.4 nM) [128]. Ku can also bind with high affinities to DNA duplex telomeric sequences with TTAGGG repeats [129,130]. The presence of the G-quartet sequence in 3′ and 5′ of DNA does not prevent Ku from binding with high affinity. It has been shown that the paradoxical presence of the main end-joining initiator Ku at telomeres ensures protection against aberrant joining by an alternative pathway [131].The use of knockdown mice for all shelterin proteins also highlighted that the presence of Ku at mammalian telomeres is associated with the protection against alternative nonhomologous end-joining, and homologous-directed repair [132]. It was shown that Ku interacts with the RNA component of the human telomerase (hTR) in vitro and in a cellular context [133]. The interaction is mediated by 47 nt in 3′ of hTR in a manner reminiscent of the interaction between yeast Ku and the stem loop of TLC1, though there is no sequence similarity between hTR and TLC1.

## 18. Ku Would also Be Able to Bind Internal DNA Sequences

Ku was also proposed to bind at DNA in the absence of DNA ends. Giffin et al. used micro-circles DNA containing a NRE1 (negative regulatory element 1) sequence, a 14 bp DNA sequence element. NRE1 is in the long terminal repeat (LTR) of mouse mammary tumor virus (MMTV) that is important for repressing inappropriate viral expression. Ku presents a strong affinity for the micro-circles that contain this NRE1 motif [134]. The micro-circles are resistant to several nucleases to exclude the possibility that Ku binds to nicks, to ends, or to structural features in the DNA. The molecular basis of the interaction of Ku on internal DNA motifs with a sequence specificity is not well understood. It is proposed that the *C*-terminal SAP domain of Ku70 may contribute with some DNA binding properties observed for this domain, though only a moderate affinity has been reported for this SAP domain [48,135].

## 19. Post-Translational Modifications of Ku Regulate Its Activities

Ku bears several post-translational modifications (PTM) that can potentially regulate its DNA recognition activities. Lees-Miller’s laboratory showed that Ku is phosphorylated by DNA-PKcs in vitro at positions Ser6 of Ku70 and Ser 577, Ser 580, and Thr 715 of Ku80, though none of these positions are the SQ consensus motif for DNA-PKcs [4,136]. A form of Ku with mutations at these five positions in alanine proved to be fully able to complement the radiation sensitivity, suggesting that the DNA-PK-dependent phosphorylation of Ku70/80 is not required for c-NHEJ [137]. Another study proposed a role for the phosphorylation of Ku on its dissociation from DSBs in a cell [53]. Using laser-generated DNA damages and live cell microscopy, an increased retention of Ku at DSBs was observed in the presence of a broad-spectrum PI3K kinase inhibitor (wortmannin). A knowledge-based approach was used to design a Ku70 mutant with Asp mutations on five potential phosphorylation sites (mutant 5D). These positions are located in the junction of the pillar and bridge regions of Ku70 (Figure 3c). This mutant has a shorter time of retention than the wild-type Ku on a DSB in cells and a weaker DNA binding affinity. A second mutant with the five positions mutated in Ala (mutant 5A), resistant to phosphorylation, has a longer retention time than the wild-type Ku. Analyses of post-translational modifications (PTMs) on the Ku heterodimer by high-resolution 2D-gel electrophoresis and the mass spectrometry of chronic lymphocytic leukemia (CLL) proteins allowed the identification of the phosphorylation of Ser27 and Ser33 of Ku70 in the resistant form of CLL [138]. These positions are located in the *N*-terminal region of Ku70 that is predicted as disordered and not visible in the crystal. These phosphorylations promote fast but unfaithful DNA repair in cancer cell lines. Sumoylation has been characterized in the *C*-terminal region of budding yeast Ku70 and shown to strengthen the association with DNA [139,140,141]. Site-specific mapping of the human SUMO proteome identified sumoylation on both Ku70 and Ku80 subunits [142].

Other PTMs of Ku have been reported. In the presence of DNA and NAD+, PARP-1 can poly(ADP-ribosyl)ate itself and Ku70/80. EMSA showed that the poly-(ADP-ribosyl)ation of Ku70/80 decreases the DNA-binding affinity of Ku [143]. In line with this observation, Hochegger et al. reported that Parp-1 inhibits Ku from binding to DSB, thereby allowing access of the HR machinery [144]. The acetylation of Ku has been shown to regulate the interaction of Ku with Bax, which suppress apoptosis by sequestering Bax from mitochondria [145,146]. It is suspected that it is the less abundant cytoplasmic pool of Ku that is responsible for Bax sequestration. This role of Ku is mediated by acetylation on eight lysines of Ku70 by either the CREB-binding protein (CBP) or P300/CBP-associated factor pCAF: Five in its C-terminus (Lys539, Lys542, Lys544, Lys553, and Lys) and three in its *N*-terminal DNA binding domain (Lys317, Lys331, and Lys338). The acetylation of Lys539 and Lys542 has been found to be critical for the regulation of Bax-mediated apoptosis [147]. Ku acetylation also disrupts Ku–p53 mRNA interactions, leading to a relief of Ku-mediated translational repression of the p53 mRNA after genotoxic stress [148].

## 20. Regulation of Ku Activities by Small Molecules

The interaction between Ku and DNA is dependent on reduced cysteines, and alkylation with *N*-ethylmaleimide prevents the interaction with DNA [54]. Similarly, the diamide treatment of Ku disrupts the Ku-DNA binding interaction, and this was abrogated by dithiothreitol treatment, demonstrating a reversible redox effect [149]. Hydroxyethyl disulfide also causes an enhanced radiation sensitivity and a direct inhibition of DNA end-binding activity of the Ku heterodimer by oxidation of its cysteine residues [150]. The analysis of the crystal structure showed that a Cys residue of Ku80 is located in the ring and could contribute to the impact of an alkylating agent (Figure 3c). An endogenous metabolite, inositol-6-phosphate (IP6), was shown to bind Ku heterodimer and stimulate the c-NHEJ pathway [151,152,153]. Molecular modeling and site-directed mutagenesis suggest an interaction site for this highly negatively charged molecule on a basic pocket of Ku at the interface between the two subunits [154]. Finally, some studies characterized interesting Ku inhibitors. Weterings et al. identified several potential inhibitors by computational screening and characterized a low micro-molar inhibitor that disrupts the binding of Ku70/80 to DNA substrates [55]. Recently, Gavande et al. described the discovery of highly potent and selective DNA-PK inhibitors that block the Ku70/80 heterodimer interaction with DNA [56] (Figure 3f).

## 21. A Well-Conserved Gene along Evolution

DSB repair factors equivalent to Ku70/80 have been identified and characterized in several bacteria [155,156], fungi [112,157,158,159], and bacteriophages [155,156,160]. Some NHEJ-like genes have also been identified in archaea, and a complete c-NHEJ system, comprising Lig, Pol, PE, and Ku, was characterized in the archaeon *Methanocella paludicola* (Mpa) [161]. Nenarokova reported frequent losses of Ku70 and Ku80 genes in several lineages of parasitic protists like human parasitic protists *Trypanosoma* spp., *Plasmodium* spp., and *Encephalitozoon cuniculi*. Multiple sequence alignments show that the core region of Ku is well conserved in most species. The similarity between the eukaryotic and bacterial Ku proteins indicates a likely common ancestral gene. The *C*-terminal region of Ku80 that is involved in the interaction with DNA-PKcs is conserved mainly in multicellular organisms that have a DNA-PKcs gene. It is absent, for example, in *S. cerevisae* that has no identified DNA-PKcs.

In silico analyses of microbial genomes identified putative Ku genes in several prokaryote genomes [155,162] (Aravind and Koonin, 2001; Doherty et al., 2001). When bacterial NHEJ was studied in prokaryotes, a Ku homodimer was found to bind DNA ends and to recruit LigD, which carries nuclease, polymerase, and ligase activities [163]. Intriguingly, some species comprise up to four orthologues of Ku and Lig, which carry out different NHEJs under specific stress conditions [164]. The bacterial Ku homologs (∼30–40 kDa) are smaller than their eukaryote counterparts. They comprise the central core of Ku70 and Ku80, and they lack the α/β *N*-terminal region and the *C*-terminal region of both subunits. No crystal structure of bacterial Ku has been available up until now but multiple sequence alignments suggest that the bacterial Ku core region shares the same fold as that of eukaryotic Ku. The mycobacterial Ku forms homodimers, binds DNA ends, and can thread on DNA, as observed in eukaryotes [165]. The c-NHEJ in many prokaryotes is performed by a two-component system constituted by the Ku homodimer and a multifunctional DNA ligase (LigD). The interaction between Ku and LigD involves the *C*-terminal region of Ku. Additional studies on Ku from *Bacillus subtilis* have revealed that the *C*-terminal region of bacterial Ku is important for Ku threading on DNA and its synapse properties [166]. Using single-molecule DNA analysis, it was shown that the *Bacillus subtilis* Ku homodimer alone, contrary to human Ku, forms a ∼2 s synapsis between blunt DNA ends that is increased to ∼18 s upon addition of LigD. These synaptic properties are dependent on the *C*-terminal arms of Ku [167]. The bacterial Ku was also used as a tool to show that blocking DNA ends affects end resection and HR in mammalian cells [168].

Original properties of Ku were reported in ciliates like *Paramecium tetraurelia.* These organisms have massively programmed genome rearrangements (PGR) during the development of their somatic nucleus with the precise elimination of at least 45,000 germline sequences (internal eliminated sequences, IESs). IES excision proceeds through a cut-and-close mechanism mediated by a domesticated transposase, PiggyMac, essential for DNA cleavage, and the c-NHEJ pathway. The genome of *Paramecium tetraurelia* encodes for two Ku70 and three Ku80 paralogs. The Ku70a and Ku80c paralogs are produced during sexual processes. The Ku70a/80c heterodimer co-purifies with PiggyMac and is essential for the licensing of its endonuclease [169]. Recently, immunofluorescence microscopy and high-throughput DNA sequencing revealed that Ku80c stably anchors the PiggyMac (Pgm) endonuclease through an interaction on the α/β domain of Ku80c [170]. The ciliate model suggests a tight coupling between DSB introduction and repair with a central role of Ku [171].

## 22. Conclusions

In conclusion, several important issues regarding the molecular mechanism of Ku are still unresolved. It is still unclear to what extent Ku is sensitive to the diversity of structures and chemistry of the DNA ends that are generated during accidental or programmed DSB;.The prominent role of Ku at telomeres are not clearly understood. It is not clear how Ku interacts with the other initial responders to DSB including MRN (Mre11-Rad50-Nbs1 complex) and PARP enzymes (Poly [ADP-ribose] polymerases). And additional studies are needed to characterize the functions of Ku that are conserved in the different organisms and those that are unique to some organisms.

Ku70/80 recognizes a large variety of DSBs with different chemistries and structures of the DNA ends. The heterodimer is one of the first players to be on the DSB site together with MRN and PARP-1, and it contributes in humans to orient DSB repair predominantly toward the c-NHEJ pathway. Despite important data on the molecular mechanism of Ku in vitro and in cells, additional studies are needed to further document how it interacts with various DNA and RNA ends and with DNA/RNA hybrids. It will be central in the future to understand how Ku interactions with its partners regulate its threading capacity in cells during the recognition of the ends and during the downstream steps of the c-NHEJ. The role of Ku beyond the c-NHEJ pathway in telomere maintenance, replication fork arrest/restart, and in the cytoplasm will also require further analyses. Finally, Ku is an important pharmaceutical target to modulate the c-NHEJ pathway [172]. The design of potent inhibitors of Ku-DNA or Ku-protein interactions will be central to characterize new molecules that may serve as a radiosensitizer or to improve genome editing.
